# Involvement of ACE2/Ang-(1-7)/MAS1 Axis in the Regulation of Ovarian Function in Mammals

**DOI:** 10.3390/ijms21134572

**Published:** 2020-06-27

**Authors:** Kamila Domińska

**Affiliations:** Department of Comparative Endocrinology, Medical University of Lodz, Zeligowskiego 7/9, 90-752 Lodz, Poland; kamila.dominska@umed.lodz.pl; Tel.: +48-426393180

**Keywords:** RAS, angiotensin-(1-7), MAS, ACE, ovary, folliculogenesis, polycystic ovary, hyperstimulation, ovarian cancer

## Abstract

In addition to the classic, endocrine renin-angiotensin system, local renin-angiotensin system (RAS) has been documented in many tissues and organs, including the ovaries. The localization and functional activity of the two opposing axes of the system, viz. ACE1/Ang II/AT1 and ACE2/Ang-(1-7)/MAS1, differs between animal species and varied according to the stage of follicle development. It appears that the angiotensin peptides and their receptors participate in reproductive processes such as folliculogenesis, steroidogenesis, oocyte maturation, and ovulation. In addition, changes in the constituent compounds of local RAS may contribute to pathological conditions, such as polycystic ovary syndrome, ovarian hyperstimulation syndrome, and ovarian cancer. This review article examines the expression, localization, metabolism, and activity of individual elements of the ACE2/Ang-(1-7)/MAS1 axis in the ovaries of various animal species. The manuscript also presents the relationship between the secretion of gonadotropins and sex hormones and expression of Ang-(1-7) and MAS1 receptors. It also summarizes current knowledge regarding the positive and negative impact of ACE2/Ang-(1-7)/MAS1 axis on ovarian function.

## 1. Introduction

Ovaries are female gonads responsible for the production of sex cells (oocytes) and the synthesis of hormones necessary for regulation of reproductive functions. The surface of the ovary is covered by a single layer of cuboidal epithelium, with the fibrous tunica albuginea tissue located underneath. The inside of the ovary is distinctly divided into an outer cortex and an inner medulla. The medulla contains blood vessels, lymphatic ducts, and nerve fibers surrounded by loose connective tissue. The cortex contains ovarian follicles, embedded in a cellular connective tissue stroma [1,2]. At any time the ovary contains follicles in many phases of development.

During folliculogenesis, the primordial follicles undergo a series of critical histological and hormonal changes, in which cell proliferation, and the differentiation and development of local blood supply, play a key role. These processes are regulated by gonadotropins (LH and FSH), sex steroids, and peptide hormones. Local peptide regulators act on the autocrine and paracrine pathways and retain a high degree of autonomy despite being subject to the overriding influence of gonadotropins [2,3].

The role of the local renin-angiotensin system (RAS) in the ovary has been documented, but its physiological role in the female reproductive process remains unclear. One of the better known active peptides of the RAS is angiotensin-(1-7), or Ang-(1-7) for short. The biological effects of Ang-(1-7) are typically antagonistic to those of Ang II, an octapeptide that was until recently considered to be the only active peptide in RAS.

Changes in the activity of the local ACE2/Ang-(1-7)/MAS1 pathway can result in fertility problems through the induction of ovarian diseases [4,5,6,7]. This review article presents positive and negative effects of Ang-(1-7) and its receptors on ovarian biology. It summarizes the expression, localization, metabolism, and activity of selected components of the RAS in the ovaries of different animal species, examines the relationship between the secretion of gonadotropin or sex hormones, and the expression of Ang-(1-7) and MAS receptors, and finally, discusses the potential for antihypertensive drugs in improving the function of the ovary and treating associated conditions. 

## 2. Angiotensin-(1-7) Synthesis and Metabolism

**Figure 1:**
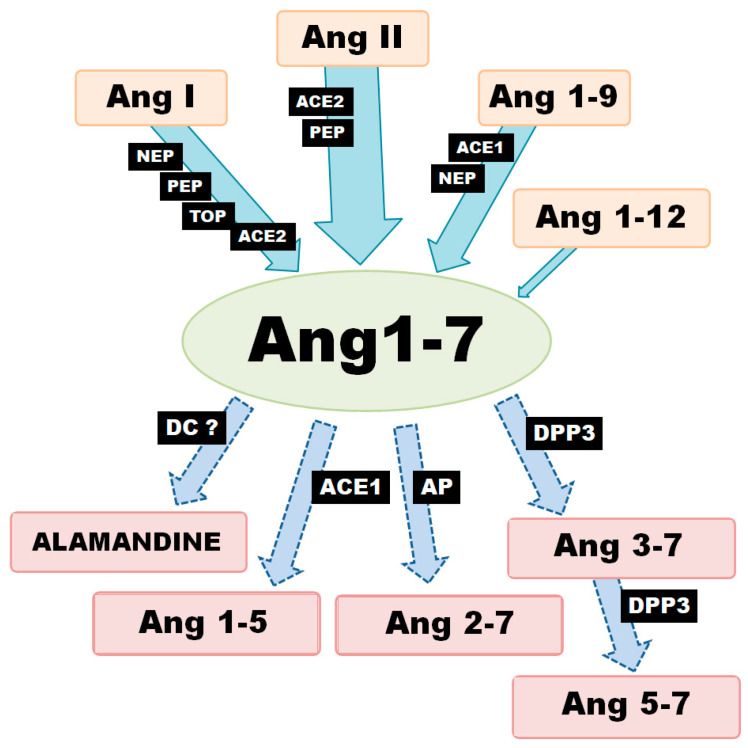
Various enzymatic pathways of Ang-(1-7) synthesis and degradation (ACE1—angiotensin converting enzyme-1; ACE2—angiotensin converting enzyme-2; AP—aminopeptidase; DC—decarboxylase; DPP3—dipeptidyl peptidase-3; NEP—neutral endopeptidase; PEP—prolyl endopeptidase; TOP—thimet oligopeptidase).

Generally speaking, there are three metabolic pathways which are responsible for the local production of Ang-(1-7) (Figure 1). The first involves the hydrolysis of Ang I by several aminopeptidases (AP): prolyl endopeptidase/prolyl oligopeptidase/angiotensinase C (PEP/POP/AC), neutralendopeptidas/neprilysin/membrane metallo-endopeptidase (NE/NEP/MME), thimet oligopeptidase (TOP). The second is associated with the cleavage of the Pro7-Phe8 bond in Ang II by endopeptidases or carboxypeptidases (CP). The main enzyme of this pathway, angiotensin-converting enzyme 2 (ACE2), can also remove the amino acid leucine from the C-terminus of Ang I to release Ang-(1-9). The generated nonapeptide can be subsequently hydrolyzed to Ang-(1-7) via either neprilysin or angiotensin-I-converting enzyme (ACE1). However, Ang-(1-9) is a 6-fold better substrate for NEP than ACE1 (kcat/Km: 3.7 × 10^5^ M−1·s−1 vs. 6.8 × 10^4^ M^−1^·s^−1^) [8,9].

In turn, ACE2 demonstrates 400-fold greater catalytic activity of with Ang II as a substrate than Ang I (kcat/Km: 2.2 × 10^6^ vs. 3.3 × 10^4^ M^−1^·s^−1^) [8]; (kcat/Km: 1.9 × 10^6^ vs. 4.9 × 10^3^ M^−1^·s^−1^) [10]. It can also hydrolyze Ang II to Ang-(1-7) 10 times more efficiently than PEP and 600 times more so than prolyl-carboxypeptidase (PRCP) [11]. These results suggest that the primary role for ACE2 is the conversion of Ang II to Ang-(1–7); it is worth noting that Ang-(1-7) and Ang-(1-9) are not cleaved by ACE2. In contrast, ACE2 appears to have a much more restricted distribution pattern than ACE1. However, recent studies suggest that ACE2 is more widespread in than first thought, being found in the heart, endothelial cells and vascular smooth muscle cells, gastrointestinal tract, kidney, brain, lung, testis and ovary tissue. In addition, Ang-(1-7) can also be created directly from Ang-(1-12), although this is definitely not the main pathway for its formation [12].

Ang-(1-7) is not the final product of the RAS and is metabolized to shorter peptides, such as Ang-(1-5) by ACE or to Ang-(3-7) and Ang-(5-7) by dipeptidyl peptidase 3 (DPP3). DPP3 first cleaves the Arg2–Val3 bond of Ang-(1-7) to generate Ang-(3-7) and the dipeptide Arg1-Asp2. Ang-(3-7) is then very rapidly cut at Tyr4-Ile5 to form Ang-(5–7) and the dipeptide Val3–Tyr4. It is worth noting that DPP2 demonstrates higher catalytic activity with Ang-(3-7) as a substrate than Ang1-7 (kcat/Km: 7.9 × 10^5^ M−1·s−1 vs. 0.6 × 10^5^ M−1·s−1) [13]. Furthermore, the enzymatic decarboxylation of Ang-(1-7) leads to the formation of alamandine (Ala1-7), a recently identified component of RAS. The two peptides differ structurally: the Asp in the N-terminal site of Ang-(1-7) is changed to Ala in alamandine [14,15]. To summarize, local Ang-(1-7) concentration depends on the activity of a range of production and degradation pathways, as well as activity of various enzymes at the tissue level.

## 3. The Receptors Involved in Ang-(1-7) Activity

**Figure 2:**
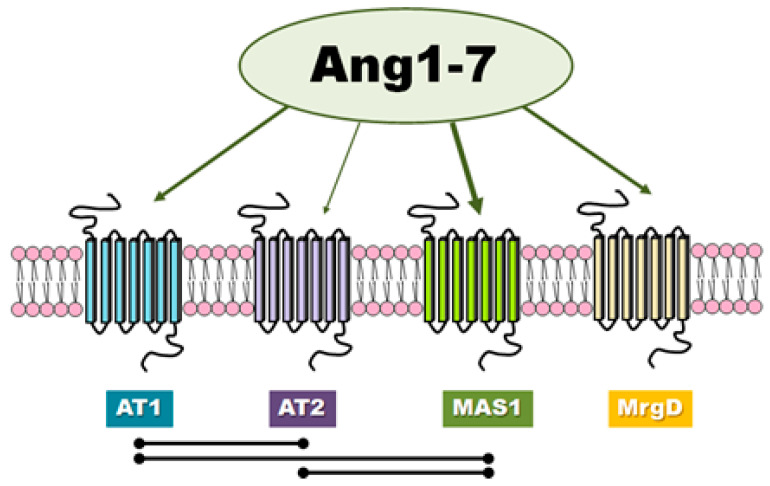
The receptors involved in Ang-(1-7) activity and the mutual interactions between them.

There is evidence in the literature that classic angiotensins receptors can mediate the effects of Ang-(1-7) although this heptapeptide displays only modest affinity at both AT1 and AT2 receptors (Figure 2). Teixeira et al. [16] noted that Ang-(1-7) has 100 times lower affinity for the AT1 receptor than Ang II (200 nM vs. 2 nM). In general, it is worth noting that the results of different authors are not consistent and have presented both high [17] and low values [18,19,20] of affinity of this peptide to AT1. Interestingly, unlike Ang II, Ang-(1-7) does not activate canonical G protein signaling. 

Ang-(1-7) was not found to influence AT1 receptor-mediated activation of Gaq or Gai, and appears to behave like a competitive inhibitor of Ang II/AT1-induced G protein-mediated signaling pathways. Furthermore, it seems that Ang-(1-7) also behaves as a strong, partial, endogenous agonist for AT1 by recruiting and activating β-arrestins (pEC50: β-arrestin 1 = 6.38, β-arrestin 2 = 6.56; Emax: β-arrestin 1 = 67.8%, β-arrestin 2 = 87.7%) [16].

It is well known that β-arrestins are involved in the desensitization, internalization, and recycling of G protein-coupled receptors (GPCRs). However, the latest research indicates that these multifunctional adapter proteins not only mediate the inhibition of GPCRs but also participate in downstream signaling independent of G-protein activation [21,22,23]. Teixeira et al. [16] report that Ang-(1-7) allows phosphorylation of ERK1/2 through a β-arrestin-dependent mechanism.

In case of AT2, Ang-(1-7) (IC50 = 2.46 × 10^∗7^ M) demonstrates nearly 500-fold less affinity than Ang II (IC50 = 5.22 × 10^∗10^ M) [20]. Interestingly, Ang-(1-7) was found to be 40 times more selective for angiotensin receptor type 2 than type 1, while Ang II was only 15 times more selective [20]. The fact that the biological effects of Ang-(1-7) can be inhibited by use of the AT1 or AT2 receptor antagonists, such as losartan and PD123319, suggest that Ang-(1-7) can act as an endogenous ligand for classic angiotensin receptors [24,25]. In contrast, it has been proposed that PD123319 may block other receptors for Ang-(1-7) described below [26].

Ang-(1–7) has also been found to serve as a ligand for the MAS1 proto-oncogene, G protein-coupled receptor (Figure 2). MAS1 is a constitutively active GPCR which undergoes endocytosis upon stimulation with Ang-(1-7) [27]. The assumption that Ang-(1-7) can associate with MAS1 was based mainly on observations that very many of Ang-(1-7) effects are lost in MAS1 knockout animals, MAS1-deficient tissues or used MAS1-specific agonists (e.g., A-779) [27,28,29,30]. However, there is no unequivocal pharmacological evidence that Ang-(1-7) interacts directly with MAS1.

Recent studies indicate that Ang-(1-7) does not initiate detectable signaling through recombinant MAS1 in transfected HEK293 cells: it did not activate signaling through the Gq and Gi family of G proteins, it did not initiate phosphorylation of Akt and Erk1/2, nor did it initiate β-arrestin recruitment by MAS1 [31].

It has also recently been postulated that another distinct Ang-(1-7) receptor may also exist which is not sensitive to A-779. For example, MrgD has been proposed as another novel receptor for Ang-(1-7) that can regulate its physiological function (Figure 2) [15,27]. However, the correct interpretation of experimental results is complicated by the lack of current knowledge regarding receptor mechanisms of signal transduction of the Ang-(1-7)-activated.

## 4. Localization and Level of Ang-(1-7) Axis Components in the Ovary

The local ACE2/Ang-(1-7)/MAS1 axis functions in the ovaries of mammals, including humans. However, the level and activity of the individual components of the RA system are dependent on the animal species and experimental model [32] (Table 1)

### 4.1. In the Rat Ovary

The rat ovary contains a moderate level of prorenin and renin activity. The renin is produced from an inactive precursor form, prorenin, by cleavage of the N-terminal 43 amino acid polypeptide. This protolithic enzyme initiates the renin-angiotensin system via the conversion of angiotensinogen to angiotensin. Angiotensinogen mRNA expression has also been observed in rat ovary, and the precursor of angiotensin peptides has been detected in granulosa cells, with lower levels being found in stroma and luteal cells [34,36]. Honorato-Sampaio et al. [33] also observed ACE2 mRNA expression in rat ovarian samples, suggesting local production of angiotensin-(1-7).

Ang-(1-7) has been identified in the theca and interstitial cells of rat oocytes, and in the stromal cells of immature unstimulated ovaries; however, the granulosa cells of antral and preovulatory follicles were negative for Ang-(1–7) [35]. Immunoreactivity to both heptapeptide and MAS1R has also been observed in rat ovaries, especially in interstitial and theca cells. Interestingly, these two types of cells are the main source of androgens in rabbit and rat ovaries [33,49,50].

The interactions between granulosa and theca interstitial cells play an important role in facilitating maximal production of estrogen. Briefly, the progesterone synthesized by granulosa cells is utilized by the theca interstitium to form androgens, which are then aromatized into estrogens by the granulosa cells [51]. Both subtypes of classic angiotensin receptors have been noted in the rat ovary; however, while the AT1 receptor is expressed in healthy follicles, AT2 is observed only in the granulosa and theca cells at the stage of the follicular atresia [7,36]. Follicular atresia is a complicated process of apoptosis in the ovary (less often autophagy or paraptosis) to regulate the number of follicles in the developing pool. The process limits the number of ovulations and can occur in all phases of follicular development [52].

### 4.2. In the Rabbit Ovary

In rabbits, Ang-(1–7) immunoreactivity has been recorded in both immature ovaries and preovulatory follicles. In the former, it is present in the stroma, and more intensely in clusters of interstitial cells, and among oocytes from primordial, primary, and secondary follicles. In the latter, immunoreactivity was observed in the granulosa cells of both the antral and preovulatory follicles. In the stroma the intensity of Ang1-7 was lower. A similar localization was observed for the MAS receptor in the rabbit ovary, but not within the granulosa cells present in the oocytes of the primordial, primary, and secondary follicles [37].

Rabbit ovaries also expressed classic angiotensin receptors. The AT1 receptor was found to be present in theca and stroma cells, and AT2 was observed in the granulosa cells of preovulatory follicles [39]. However, other studies reported only AT1 to be present [36,40].

Evidence suggests that Ang1-7 is produced locally in rabbit ovary. Zappulla and DesGroseillers [38] report the presence of NEP, an enzyme that can convert polypeptide Ang I or Ang-(1-9) into Ang-(1-7) in ovarian tissue. Nprilysin was localized at the surface of the follicular cells at various stages of the folliculogenesis procedure: from the primary to the Graafian follicles. Immunofluorescence signal of NEP has been observed in the granulosa and corona radiata cells, but not on the theca cells. Furthermore, epithelial cells surrounding the ovary as well as endothelial cells of blood vessels also expressed the neutral endopeptidase. Interestingly, the expression of NEP was repressed in the stroma of the atretic follicles when they entered regression [38]. Follicular atresia affects all stages of follicular development and is important for understanding the many and varied conditions of infertility.

### 4.3. In the Ewe Ovary

In ewe antral follicles and mature luteal cells, the theca cell layer presents strong immunoreactivity for Ang-(1-7) and ACE2, while in the antral follicles, the granulosa cells showed weak labeling. The stromal cells also exhibited positive labeling for both Ang-(1-7) and ACE2. In addition, the presence of heptapeptide was detected in follicular fluid (FF) but its concentration was almost 10 times lower than that of Ang II (37.3 ± 3.8 vs. 338.0 ± 64.9 pg/mL) [41].

### 4.4. In the Bovine Ovary

Tonellotto dos Santos et al. [44] showed existence of the ACE2/Ang-(1-7)/MAS1 axis in periovulatory follicles in the in cattle. First, it was found that the concentrations of Ang-(1-7) in follicular fluid obtained from preovulatory follicles has been more than 30 pg/mL. Next, the relative mRNA expression of MAS, ACE2, NEP, and PEP in granulosa and theca cells was noted, however at different levels. The level of MAS and ACE2 was higher in granulosa cells, while there were no differences between expression of NEP and PEP in both groups of ovarian cells [44]. The bovine ovaries expressed also classic angiotensin receptors. The angiotensin receptor type 1 was dominant in the stroma. The autoradiography presented also the intense AT2 binding in the theca cells of the majority of antral follicles and follicular cysts. The granulosa layer did not show any marking point of AT2. The AT2 receptor, but not AT1, showed cyclic changes and the receptor density was higher in value in estrus than in diestrus [45].

Changes have also been reported in MAS, PEP, and ACE2 mRNA expression in the granulosa of the largest (F1) and second largest (F2) follicles during follicular wave development, but not in the theca cells [42]. The mRNA level of ACE2 and PEP was elevated during and after the establishment of follicular deviation in follicle F1 or F2, but to different degrees. In the case of the MAS receptor expression was upregulated in the granulosa cells of dominant follicle and future largest subordinate follicle after induced follicular atresia or after follicular deviation, respectively. It was found that the thecal cells are the major source of prorenin in bovine ovarian follicles, rather than the granulosa cells [42]. Furthermore, the highest level of prorenin activity was observed in fluid taken from atretic follicles [36,43].

In cattle, ovarian follicles develop in a wave-like pattern. Over a period of 7–10 days, the cattle follicles pass through different stages of maturity, such as emergence, selection, deviation, dominance and atresia, or ovulation. Deviation in follicle diameter is observed in cattle: the largest follicle (dominant follicle) undergoes continued growth during follicular waves, while the smaller follicles so-called subordinate follicles experience reduced or even absent growth. There is evidence that subtle changes in hormonal milieu regulate the pattern of bovine waves and control the phases of the folliculogenesis [53,54]. These results suggest that Ang1-7 may influence follicular deviation in cattle and that MAS may be a receptor which can play an important role in follicular atresia and the establishment of follicular dominance [42].

### 4.5. In the Human Ovary

In humans, the ovary is the most important source of plasma prorenin [36]. As we have already mentioned, prorenina is a direct precursor of renin, a proteolytic enzyme that catalysis the conversion of angiotensinogen to Ang I. The Ang I can be hydrolyzed to Ang1-7. However, the opposite to Ang II, concentration of Ang1-7 in follicular fluid was significantly lower compared with the level of this heptapeptide in the plasma (191 vs. 407 pg/mL). Ang-(1-7) has a short half-life in plasma and is rapidly degraded. Ang II can also be hydrolyzed into Ang-(1-7) by angiotensin-converting enzyme 2 (ACE2), which is expressed in the human ovary [47]. This finding also suggests that Ang-(1-7) is produced locally in human ovaries. The stromal cells of the follicle have been found to be positive for Ang-(1–7), ACE2, and the MAS receptor; however, those of ovaries from postmenopausal women displayed less intense Ang-(1-7) and MAS staining. 

The menopausal ovary consists primarily of the stromal cells. These have a steroidogenic capacity for producing androstenedione in conditions of high gonadotropin secretion, which is aromatized to estrone by peripheral tissue [47]. ACE activity in the ovary was significantly higher in postmenopausal women than premenopausal women (1.35 vs. 0.65 nmol/mg*min). However, no such relationship was observed for ACE activity in serum. In patients with active ovaries, ovarian ACE activity was similar in the pre- and postovulatory phases [55].

As anticipated, ACE, ACE2, and MAS mRNA has also been found in luteinized granulosa cells [47,48]. In addition, MAS mRNA levels in isolated granulosa cells correlated with the number of mature oocytes. Regarding the Ang-(1-7) and MAS receptor, the primary follicles of the granulosa layer demonstrated moderate labelling, while the secondary follicles demonstrated intense labelling; in addition, weak Ang-(1-7) staining and variable (medium to high) immunoreactivity was observed for MAS in the granulosa layer of preovulatory follicles. The theca cells of secondary follicles intensively stained for Ang-(1–7) and MAS, while those of preovulatory follicles were negative for both [7,47]. The cytoplasm of both small and large luteal cells in the corpus luteum strongly stain for

Ang-(1-7). Mas immunoreactivity was not observed in the cells of the corpora lutea [47].

Such variation in the ovarian renin-angiotensin system between species reflect its different physiological roles. The changes in the level and localization of RAS elements over different phases of the ovarian cycle can be attributed to the fact that gonadotropin control of ovarian production FSH and LH plays an important role in the local function of this local peptide hormone system.

## 5. The Relationship between Gonadotropins, Sex Hormones, and ACE 2/Ang-(1-7)/MAS1 Axis in the Ovary

Theca interna expressing LH receptors and granulosa cells expressing FSH receptors play an important role in follicular development. The pituitary gonadotropins initiate the ability of the ovarian cells to synthesize and secrete steroid hormones. LH act on the theca interna cells of the ovaries to stimulate the synthesis and production of androgens such as androstendion and testosteron. Follicle-stimulating hormone (FSH) induces estrogen biosynthesis to form androgens. The granulosa cells produce also pregnenolone and progesterone [2,3]. The regulation of the secretion of steroid hormones and peptide hormones such as Ang-(1-7) by LH and FSH is not fully understood. However, the studies indicate that Ang-(1-7) is a mediator of gonadotropin functions in the ovulatory cascade [7] (Table 2).

### 5.1. In the Rat and Mouse Ovary

The components of ACE2/Ang-(1–7)/MAS1 axis are fully expressed in the rat ovary and are regulated by pituitary gonadotropic hormones [35]. Follicle-stimulating hormone stimulates renin mRNA expression in the ovaries of rats [36]. Pereira et al. [35] showed enhancement of Ang-(1-7) and MAS1 expression in the ovarian theca-interstitial cells in immature female Wistar rats after eCG treatment. Although ACE2 mRNA expression levels increased in ovaria homogenates from gonadotropin (eCG)-treated rats, ACE2 activity was lower. Decreased activity was also observed in the case of the NEP enzyme, while PEP activity was higher compared with control animals. The authors suggest that Ang-(1-7) and Ang II play complementary roles in ovarian steroidogenesis. In the theca-interstitial cells, the Ang-(1-7)/MAS1 axis modulates androgen synthesis while in granulosa cells, Ang II promotes the conversion to estradiol [35].

In contrast, Honorato-Sampaio et al. [33] noted that the stimulation of rat ovaries with hGC results in 4.5-fold greater level of ACE2 mRNA compared with controls, but no increased MAS1 receptor expression. In addition, stronger Ang-(1-7) and ACE2 immunoreactivity was found in the preovulatory follicles after 4-h incubation with luteinizing hormone (LH). The LH stimulated testosterone (T), and decreased progesterone (P4) levels, but did not influence estradiol (E) levels in preovulatory follicles. The Ang-(1-7) alone did not impact on sex hormone production, but specific Mas receptor blocker (A-779) reduced levels of P4 and increased T after LH-stimulation. The authors suggest that Ang-(1–7) may regulate follicular atresia by regulating the level of androgens [33]. Androgens induce apoptosis in granulosa cells [56] while progesterone prevents this process [57]. 

In rats, a study evaluated the influence Ang-(1-7) on the follicle maturation by resumption of oocyte meiosis. At the start of each estrous cycle, in response to a surge of luteinizing hormone (LH), a limited number of primordial follicles was found to resume meiosis until metaphase II. The mechanisms by which gonadotrophins induce oocyte maturation involve the participation of several paracrine and autocrine factors. The Ang-(1–7) promoted meiotic resumption of follicle-enclosed oocytes (FEOs) but only at a concentration of 10^∗8^ M. Moreover, Ang-(1-7) induced significantly lower germinal vesicle breakdown (GVB) than meiotic resumption induced by LH. The Mas inhibitor blocked the oocyte maturation non-stimulated and induced by both Ang-(1–7), and LH but to a different extent. The effect of Ang-(1–7) on meiotic resumption was similar to the effect of Ang II observed in other species [33]. 

Another study confirmed that MAS1 receptor can influence mouse fertility. Namely, Mas Knockout (Mas-KO) mice exhibited reduced litter size and decreased spontaneous but not induced ovulatory rate compared with WT mice. Although the estrous cycle duration did not differ in WT and Mas-KO mice, and histological analysis showed no obvious abnormality in Mas-deficient mice, a reduction was observed in the number of total follicles in knockout mice. The reduced follicular pool was associated with the reduction of IGF-1 mRNA levels in the ovaries [58]. Interestingly, ACE inhibitors, which also upregulate Ang-(1–7), are also associated with higher levels of insulin-like growth factor-1 (IGF-1). IGF-1 is produced by ovarian granulosa cells and regulates gonadotropin responsiveness and follicular development. As ACE inhibitors block the production of angiotensin II, which is a potent inhibitor of IGF-1 production, it was hypothesized that treatment with ACE inhibitors is associated with higher levels of IGF-1 [59]. Furthermore, expression of AT2 is upregulated by insulin-like growth factor 1. These results suggest that Mas receptor can promote follicular survival by IGF-1, which acts as an antiapoptotic factor. IGF-1 for example mediates BCL-2 upregulation, inhibits BCL-2 family members, delays caspase 9 activation, and activates PI3K-AKT signaling pathway [60,61].

### 5.2. In the Rabbit Ovary

Angiotensin-(1–7) increased the ovulatory efficiency in an isolated rabbit ovary model to the same extent as hCG. In both cases, this effect was significantly antagonized by A-779 treatment, although Mas receptor antagonist alone did not influence ovulatory efficiency. Furthermore Ang-(1–7) significantly stimulated release estradiol but not progesterone in rabbit ovaries. The levels of progesterone production changed only under the influence of hCG [37]. Inhibition of ACE did not influence progesterone production but estradiol level decreased in rabbits stimulated with gonadotropins [62]. The potential mechanism for the relationship between Ang-(1–7) and ovulatory process is still unclear. However, Viana et al. [37] suggest that Ang-(1–7) stimulates ovulation via increased prostaglandin formation or via the bradykinin-NO pathway [37].

### 5.3. In the Ewe Ovary

The angiotensin-converting enzyme inhibitor, enalapril decreases 17β-estradiol plasma concentration in ewes on 11 and 14 days after the beginning of estrous synchronization but did not influence testosterone and progesterone production. Furthermore, enalapril-induced group in day 14, showed increased P4:E2 ratio. However, this result did not translate into a reduction in embryo quality connected by premature luteinization of follicles or poor oocyte maturation [41].

### 5.4. In the Bovine Ovary

The GnRH increases the concentration of Ang-(1-7) in the follicular fluid of cattle preovulatory follicles after at least 24 h treatment. The relative mRNA expression of MAS receptor in granulosa and theca cells did not change after GnRH injection. Interestingly, in granulosa cells, ACE2 and PEP enzyme expression was initially downregulated after GnRH treatment but significantly rose near ovulation. In contrast, the mRNA level of NEP was upregulated in granulosa cells by GnRH, but statistical significance was only achieved for 12 and 24 h incubation, i.e., during the periovulatory period. The mRNA expression of all tested enzymes did not change after GnRH in theca cells. The results suggest that Ang-(1-7) can be involved in the regulatory process of ovulation in cattle [44].

### 5.5. In the Human Ovary

Women stimulated with gonadotropins during preparation for in vitro fertilization (IVF) are characterized by an elevated level of plasma Ang-(1-7). Nerveless, the Ang-(1-7) concentration in their follicular fluid is still lower than in the plasma [47]. This is probably due to the fact that this heptapeptide is very quickly metabolized in the ovary by NEP or PEP. Cavallo et al. [48] examined whether the level of Ang-(1–7) in human ovarian follicular fluid (FF) correlates with the number and proportion of mature oocytes obtained for IVF. Firstly, in patients after ovarian stimulation (COS) with human urinary gonadotropin (hCG) and/or recombinant FSH (rFSH) noted increased plasma concentrations of Ang-(1–7). Furthermore, a linear correlation between Ang-(1-7) levels in human FF and the proportion of mature oocytes was discovered. These findings indicate that FSH and hCG may mediate ovarian RAS activation [48]. Moreover, negative correlation between expression of AT1 receptor in FF granulosa cells and the number of mature oocytes was observed in women with poor ovarian reserve [63].

## 6. Ovarian Diseases Related to the ACE2/Ang-(1-7)/MAS1 Axis

The changes in the level of selected components of the renin-angiotensin systems were observed in pathological states of the reproductive tissues, including ovary. The involvement of the ACE2/Ang-(1-7)/MAS1 axis in ovarian diseases has been confirmed for polycystic ovary syndrome and ovarian hyperstimulation syndrome, as well as in ovarian cancer [64].

### 6.1. Polycystic Ovary Syndrome (PCOS)

Polycystic ovary syndrome (PCOS) is a highly prevalent endocrine-metabolic disorder characterized by elevated androgen levels (hyperandrogenism), cystic or enlarged ovaries, ovulatory dysfunction such as absent ovulation, menstrual irregularities, or amenorrhea. Furthermore, PCOS is very often associated with infertility, obesity, impaired glucose metabolism and insulin resistance, impaired lipid profile and high blood pressure, and cardiovascular diseases [65].

Some studies have demonstrated that RAS members are upregulated in women with PCOS. Women with histologic diagnosis of PCOS presented intense labeling for renin and angiotensin in both theca and granulosa cells of the large cystic follicles while in the follicles of normal ovaries immunostaining was restricted to the theca cell layer [46]. Qin et al. [66] evaluated the expression of RAS components in the endometrium in polycystic ovary syndrome (PCOS) patients. The level of mRNA expression of ACE2 enzyme and AT1, AT2, and MAS receptors was higher in the PCOS endometrium than in the control group [66]. Jaatinen et al. [67] examined serum total renin in 44 women with PCOS and found that concentration of this proteolytic enzyme is higher than healthy women, independent of BMI, age, or serum insulin [67]. On the other hand, Arefi et al. [68] did not find any significant differences between plasma renin activity in women with PCOS and controls; however, they note that both ACE activity and Ang ΙΙ levels were significantly higher in patients with this endocrine disorder [68]. Alphan et al. [69] report that obese women with PCOS have higher total renin concentrations, and these were correlated with fasting insulin levels and free testosterone level. The authors did not notice changes between aldosterone concentration and serum ACE activity [69].

The results of many studies demonstrated that Ang-(1-7) can improve the action of insulin and that it participates in the maintenance of normoglycemia. This heptapeptide enhanced insulin delivery to target tissues by vasodilation and increased blood flow and also counteracted the effects of Ang II. People with type 2 diabetes demonstrated lower circulating concentrations of Ang-(1–7). Similarly, in the case of pregnant women, higher levels of Ang-(1–7) were observed in healthy than in women with gestational diabetes [34,70].

Indeed, substantial evidence has shown that modulation of the ACE2/Ang-(1–7)/MAS1 receptor axis could improve both hemodynamic and metabolic diseases in humans. It has been shown that anti-hypertensive drugs including ARBs (angiotensin receptor blockers) and ACE inhibitors may act to prevent diabetes and lead to improvement in insulin resistance. Bonita Falkner et al. [71] noted that lisinopril treatment increases insulin sensitivity in young adults with mild hypertension [72]. Several meta-analyses have also confirmed the positive effects of AT1R and ACE inhibitors on insulin sensitivity and the progression to type 2 diabetes [70,72].

There is significant evidence from human studies and experimental work on the hyperandrogenic animal models that excess androgens working via the AR play an important role in the origin of PCOS. Approximately 60% of patients with PCOS exhibit hyperandrogenism. It is worth mentioning that excess androgens can be induced by insulin resistance and hyperinsulinemia. These changes cause reductions in sex hormone binding globulin levels, which leads to a subsequent increase in free androgens and unfavorable metabolic profiles [73]. Moreover, it has been shown that anti-hypertensive drugs that block the RAS may lead also to decreased androgen levels in women with PCOS. For example, the higher levels of plasma ACE lead to a higher level of Ang-II and steroid hormone synthesis disorders. Hacıhanefioglu et al. [74] report that lisinopril treatment reduced total and free testosterone, androstenedione, DHEAS, and 17-OHP but increased level of SHBG in hypertensive women with PCOS.

Additionally, some research suggests that the D allele of the ACE gene is associated with the formation of polycystic ovary and hyperandrogenism [64,75,76]. On the other hand, Sun et al. [77] denied a direct relationship between the I/D polymorphisms in the ACE gene and pathogenesis of PCOS. However, the occurrence of polymorphisms was associated with the steroidogenesis in the ovary, suggesting that D allele of the ACE gene plays a role in worsening the symptoms of PCOS [77].

### 6.2. Ovarian Hyperstimulation Syndrome (OHSS)

Ovarian hyperstimulation syndrome (OHSS) is considered an iatrogenic consequence of controlled ovarian stimulation (COS) by gonadotrophin. However, OHSS can also arise spontaneously, for example in pregnancy, but this is extremely rare. High levels of VEGF and the RAS seem to play an important role in the formation of neovascularization responsible for the development of OHSS. The ovarian hyperstimulation syndrome is characterized by cystic enlargement of the ovary and an increase in the permeability of the capillaries. Endothelial dysfunction leads to a fluid shift from the intravascular space to the extravascular compartments [78].

hCG causes strong activation of the RAS, evidenced by high renin activity in the follicular fluid of women with OHSS. A direct correlation has been demonstrated between plasma renin activity, aldosterone level and the severity of OHSS. The levels of prorenin and Ang II were much higher in the ascites of severe OHSS than in the pleural fluid and the plasma [79,80,81]. Morris et al. [82] reported that enalapril, an ACE1 inhibitor, decreased the incidence of OHSS in a rabbit model; however, Gul et al. [83] did not notice any significant benefits of enalapril administration in reducing the severity of OHSS symptoms such as ascites formation. On the other hand, Morris et al. [82] report that ACE inhibitors increase the plasma level of estradiol in treated rabbits.

It is worth noting that estradiol is an important marker of increasing risk for OHSS. The next two research teams tried to evaluate the efficacy of combined treatment of angiotensin-converting enzyme inhibitor (ACE1) and angiotensin II receptor blocker (ARB) on preventing early OHSS in IVF patients at very high risk for this syndrome [84,85]. It is worth noting that orally administered drugs will never be first choice during treatment of OHSS due to the possible teratogenic effect in humans. Furthermore, the increased renin activity in OHSS could be only the effect and not the cause of this complication [86].

### 6.3. Ovarian Cancer (OC)

Ovarian cancer is not one of the most common cancers in women, but it is one of the deadliest because its early symptoms are usually mistaken for menstrual cramps and pain or indigestion and upset stomachs. OC, unlike most solid tumors, is distributed throughout the peritoneal cavity then metastasizes via the blood. Recent years have brought radical changes in the theory of OC etiology, challenging the classical idea that all OCs originate from the epithelium of the ovarian cell surface. It is now known that OC is a heterogeneous disease comprising several histologic types with different biological behaviors and clinicopathological characteristics. An analysis of the gene expression profile indicated a relationship between individual histological types of OC and normal epithelium. Oncological gynecologists increasingly believe that serous OC is derived from the fallopian tube or ovarian epithelium while clear-cell and endometrioid cancers may arise from the glandular cells of the cervical canal and/or endometrioid foci. The mucinous tumors of the ovary seem to have common origin with metastases from the lower gastrointestinal tract [87,88]. 

**Figure 3:**
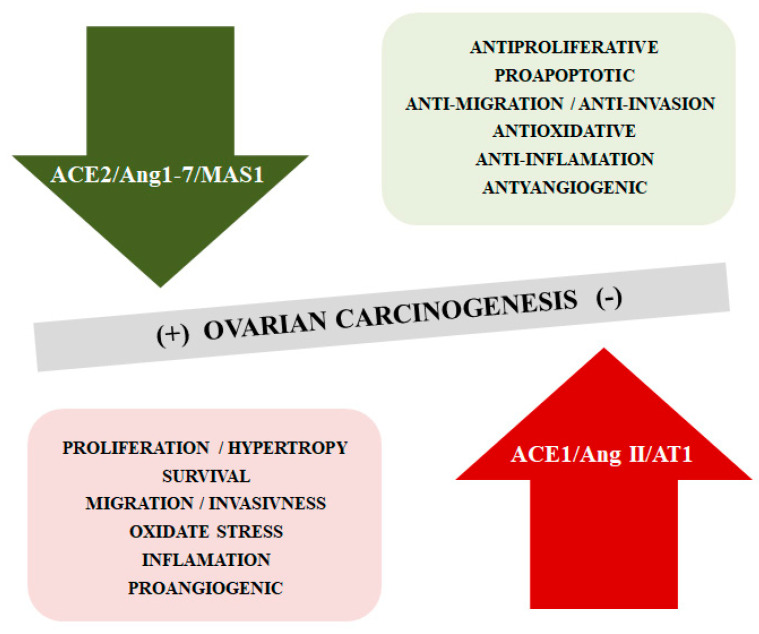
The opposite function of ACE2/Ang-(1–7)/MAS1 and ACE1/AngII/AT1 axis in the ovarian cancerogensesis.

The literature confirms changes in selected components of the RAS in different types of tumors, including OC. The mechanisms of tumorigenesis and cancer progression associated with these changes still remain unclear [89,90,91]. It has been described that the ACE2/Ang1-7/MAS1 cascade opposes the effects of the ACE1/Ang II/AT1 receptor axis, inhibiting cancer cell proliferation, reducing the migratory potential and invasiveness, limiting the formation of new blood vessels. However, it appears that the ACE2/Ang-(1–7)/MAS1 signaling axis can play both tumor-promoting and inhibiting functions depending on the type of cancer [92,93] (Figure 3).

In the patients with epithelial OC, serum ACE levels were significantly higher than controls (30.58 vs. 14.15 U/L). No differences were observed between the stage of the tumor (FIGO I–II vs. FIGO III–IV) as well as between serous and non-serous OC patients [94]. Cotter et al. [95] suggested on the basis of case report patients with metastatic ovarian carcinoma that serum ACE1 level may be a marker for ovarian germ cell tumors (dysgerminoma) and may be useful for monitoring treatment [95]. On the other hand, Correa-Noronha et al. [96] did not observe an association between the insertion/deletion (I/D) polymorphism in the angiotensin-converting enzyme (ACE) gene and epithelial OC [96].

Expression of AT1 was present in 85% of invasive ovarian carcinomas and was not dependent on the histopathologic subtype. Angiotensin receptor type 1 was localized in the cytoplasm of both normal and tumor ovarian cells. The AT1 immunoreactivity on the surface epithelium was only present for the OC cells. Furthermore, the results indicate that AT1 expression increased from benign to advanced OC [97]. Suganuma et al. [98] noted some correlation between expression of AT1 receptor and the stimulation of angiogenesis processes in OC. The VEGF expression and microvessel density was significantly higher in strongly AT1-positive OC tissues than in weakly positive or negative ones. In vitro studies on OC cell lines indicate that Ang II (10^8^–10^7^ mol/L) significantly enhanced the invasive potential AT1R-positive SKOV-3 cells but not in AT1R-negative HRA cells. Furthermore, only SKOV3 lines demonstrated significantly enhanced VEGF mRNA expression after treatment with Ang II. Both effects were inhibited by the AT1R antagonist(candesartan) but not by the AT2R blocker PD123319. Moreover, candesartan also suppressed tumor growth and progression as well as neovascularization in a mouse model in vivo [98]. Ino et al. [97] demonstrated that AT1 expression did not significantly correlate with clinicopathological factors in OC patients (e.g., histological subtype, FIGO stage, or histological grade) but was correlated with VEGF overexpression and high MVD number (tumor angiogenesis) and poor patient outcome in OC [97].

Recently Zhang et al. [99] obtained convergent results indicating that high AT1 expression was negatively correlated with survival time for grade 1 and 2 ovarian tumor patients. Ang II was again found to increase OC cell proliferation by AT1 receptor, but the results were significant only in the 3D culture model. The authors note that not only did Ang II promote multicellular spheroid (MCS) growth and metastasis of OC cells but also that Ang II and AT1 formed a positive feedback loop that enhanced OC progression. Secretion of Ang II by ovarian cells was observed in in vitro cell lines, xenograft mouse models, and patient samples. Zhang et al. [99] suggest that AGTR1 activates ERK and AKT signaling, as well as transactivation of the EGFR pathway. Furthermore, Gene Ontology (GO) analysis showed that Ang II upregulated multiple SREBP pathway-related genes and downregulated genes of JNK cascade. The authors conclude that Ang II upregulates SCD1, which promotes OC progression and metastasis by improving the lipid desaturation and relieving endoplasmic reticulum stress during the formation and growth of MCS [99]. 

Song et al. [100] demonstrated the stimulatory effects using angiotensin II type I receptor agonistic autoantibodies (AT1-AA) purified from OC patients on tumor cell migration and angiogenesis. The AT1-AA bind with high affinity to a seven-amino acid sequence on the second extracellular loop of the angiotensin receptor type 1 and activate intracellular pathways similar to Ang II. The serum AT1-AA titer in OC patients was significantly higher than in healthy control subjects (1.77 vs. 0.35). Moreover, the AT1-AA level increased with an advanced FGIO stage and grade in OC patients [100].

## 7. Conclusions

In conclusion, there is no doubt about the presence of the local renin angiotensin system in mammalian ovaries. Many research results indicate local production of angiotensins, including Ang-(1-7) in the ovary. Nevertheless, the involvement of the ACE1/Ang II/AT1 and ACE2/Ang-(1-7)/MAS1 axes in the physiology and pathology of the female reproductive system is still unclear and incomplete. RAS appears to play an important role in mediating gonadotropin function and is involved in ovarian steroidogenesis. Changes in the level of some system components during the estrus/menstrual cycle indicate the involvement of angiotensins and their receptors in follicular development and perhaps also ovulation. Interpretation difficulties arise from species differences and a complicated network of mutual dependencies in the discussed peptide hormone system. Undoubtedly, further research in this area is required to better understand the impact of RAS on female fertility and to determine its contribution to such diseases as polycystic ovary syndrome of ovarian hyperstimulation syndrome or OC.

## Figures and Tables

**Table 1:** The confirmed location of individual members of ACE2/Ang-(1-7) axis in the mammalian ovary (Ang-(1-7)—angiotensin-(1-7); ACE2—angiotensin-converting enzyme 2; NEP—neutral endopeptidase; PEP—prolyl endopeptidase; MAS1—MAS receptor; AT1—AT1 receptor; AT2—AT2 receptor).

	Elements OF ACE2/ANG1-7 Axis								
	PRORENIN, RENIN	ANGIOTENSINOGEN	Ang-(1-7)	ACE2	NEP	PEP	MAS1	AT1	AT2
**Rat** **Ovary**	→ corpora lutea but not in theca or granulosa of follicles [33]	→ granulosa cells of maturing and atretic follicles [34] → antral cell layers of maturing follicles [34] → follicular fluid taken from maturing follicles [34]	→ theca and interstitial cells of oocytes [33,35] → stromal cells of immature unstimulated ovaries but granulosa cells of antral and preovulatory follicles not [35]	no data	no data	no data	→ theca and interstitial cells of oocytes [33]	healthy follicles [36]	→granulosa and theca interna cell layers of atretic follicles [36]
**Rabbit Ovary**	no data	no data	→ stromal and interstitial cells of immature ovaries [37] → granulosa cells of antral and preovulatory follicles [37]	no data	→ granulosa and corona radiata of follicular cells [38] → epithelial cells surrounding the ovary and endothelial cells of blood vessels [38] → stromal cells but not of the atretic follicles [38]	no data	→ stromal and interstitial cells of immature ovaries [37]	→ theca and stroma cells of preovulatory follicles [39,40]	→ granulosa cells of preovulatory follicles [39]
**Ewe** **Ovary**	no data	no data	→ antral follicles and mature luteal cells, the theca and stromal cell layer [41] → follicular fluid taken from antral follicles [41]	→ antral follicles and mature luteal cells, the theca and stromal cell layer [41]	no data	no data	no data	no data	no data
**Bovine Ovary**	→ thecal cells rather than the granulosa cells of ovarian follicles [42] → fluid taken from atretic follicles [43]	→ follicular fluid taken from preovulatory follicles [44]	no data	→ granulosa and theca cells of periovulatory follicles [44]	→ granulosa and theca cells of periovulatory follicles [44]	→ granulosa and theca cells of periovulatory follicles [44]	→ granulosa and theca cells of periovulatory follicles [44]	→ stroma of periovulatory follicles [45]	→ theca cells of majority of antral follicles and follicular cysts [45]
**Human Ovary**	→ theca, stromal, luteal, and granulosa cells of ovarian follicles [36,46] → follicular fluid [36]	no data	→ stromal and granulosa cells of the follicle [47,48] → theca cells of secondary but not preovulatory follicles [47] → small and large luteal cells in the corpus luteum [47]	→ stromal and granulosa cells of the follicle [47]	no data	no data	→ stromal and granulosa cells of the follicle [47,48] → theca cells of secondary but not preovulatory follicles [47]	no data	no data

**Table 2:** Interaction between gonadotropins and sex hormones and ACE2/Ang-(1-7) axis. (FSH— follicle-stimulating hormone; LH—luteinizing hormone; T—testosterone; E2—estradiol; P4—progesterone; ACE1—angiotensin-converting enzyme 1; Ang-(1-7)—angiotensin-(1-7); ACE2—angiotensin-converting enzyme 2).

Species	Gonadotropins	FSH	LH	Ang-(1-7)	ACE1 Inhibitor
**Rat**	stimulates Ang-(1-7) and MAS1 expression in the ovarian theca- interstitial cells [35] increases ACE2 mRNA but not their activity in ovaries [33,35] increases PEP activity but inhibites NEP activity in ovaries [35]	stimulates renin mRNA expression in the ovary [36]	inreases Ang-(1-7) and ACE2 immunoreactivity in the preovulatory follicles [33]	non impact on sex hormone production [33]	reduces levels of P4 and increased T after LH-stimulation [33]
**Rabbit**	no data	no data	no data	stimulates release E2 but not P4 in ovaries [37]	not influence on P4 production but E2 level decreased after gonadotropins-stymulation [37]
**Ewe**	no data	no data	no data	no data	decreases E2 plasma concentration but not influence on T and P4 production [41] showes increased P4:E2 ratio [41]
**Bovine**	increases Ang-(1-7) concentration in the follicular fluid of preovulatory follicles [44] downregulates ACE2 and PEP expression in granulosa cells but not near ovulation [44]	no data	no data	no data	no data
**Human**	elevates level of plasma Ang-(1-7) [47,48]	increases plasma concentrations of Ang-(1–7) [48]	no data	no data	no data

## Abbreviations

Ang-(1-7)	Angiotensin 1-7
OC	Ovarian cancer
VEGF	Vascular endothelial growth factor
ACE	Angiotensin converting enzyme
LH	Luteinizing hormone
FSH	Follicle-stimulating hormone
RAS	Renin-angiotensin system
AP	Aminopeptidase
DC	Decarboxylase
DPP3	Dipeptidyl peptidase 3
CP	Carboxypeptidase
PRCP	Prolyl-carboxypeptidase
GPCRs	G protein-coupled receptors
hGC	Human urinary gonadotropin
eCG	Equine chorionic gonadotropins
EFOs	Follicle-enclosed oocytes
GVB	Germinal vesicle breakdown
IGF-1	Insulin-like growth factor 1
P4	Progesterone
E2	Estradiol
T	Testosterone
GnRH	Gonadotropin-releasing hormone
rFSH	Recombinant FSH
FF	Human ovarian follicular fluid
COS	Ovarian stimulation
IVF	In vitro fertilization
PCOS	Polycystic ovary syndrome
AR	Androgen receptor
DHEAS	Dehydroepiandrosterone sulfate
SHBG	Sex hormone-binding globulin
OHSS	Ovarian hyperstimulation syndrome
MCS	Promote multicellular spheroid
